# Supplementary material for: Investigating the influence of physiologically relevant hydrostatic pressure on CHO cell batch culture
Source: Sci Rep. 2021 Jan 8;11:162. doi: 10.1038/s41598-020-80576-8 (PMC7794228; doi:10.1038/s41598-020-80576-8)
Supplement: Supplementary file 1 — Supplementary Information 1. [file 41598_2020_80576_MOESM1_ESM.zip › S1/IgG/Feb17 60 90 standard/old/Feb17 90mmHg 3.pdf]

Data File C:\Users\P...mStation\1\Data\TK_CHO_IgG 2019-02-17 14-04-01\036-7-90mmHg Feb17.D
Sample Name: 90mmHg Feb17

===

Acq. Operator : SYSTEM   Seq. Line : 36
Sample Operator : SYSTEM
Acq. Instrument : HPLC-0039   Location : 7
Injection Date : 2/17/2019 4:34:59 PM   Inj : 1
Inj Volume : 100.000 µl
Method : C:\Users\Public\Documents\ChemStation\1\Data\TK_CHO_IgG 2019-02-17 14-04-01
\CHO_IgG_LC.M (Sequence Method)
Last changed : 2/8/2019 3:17:23 PM by SYSTEM

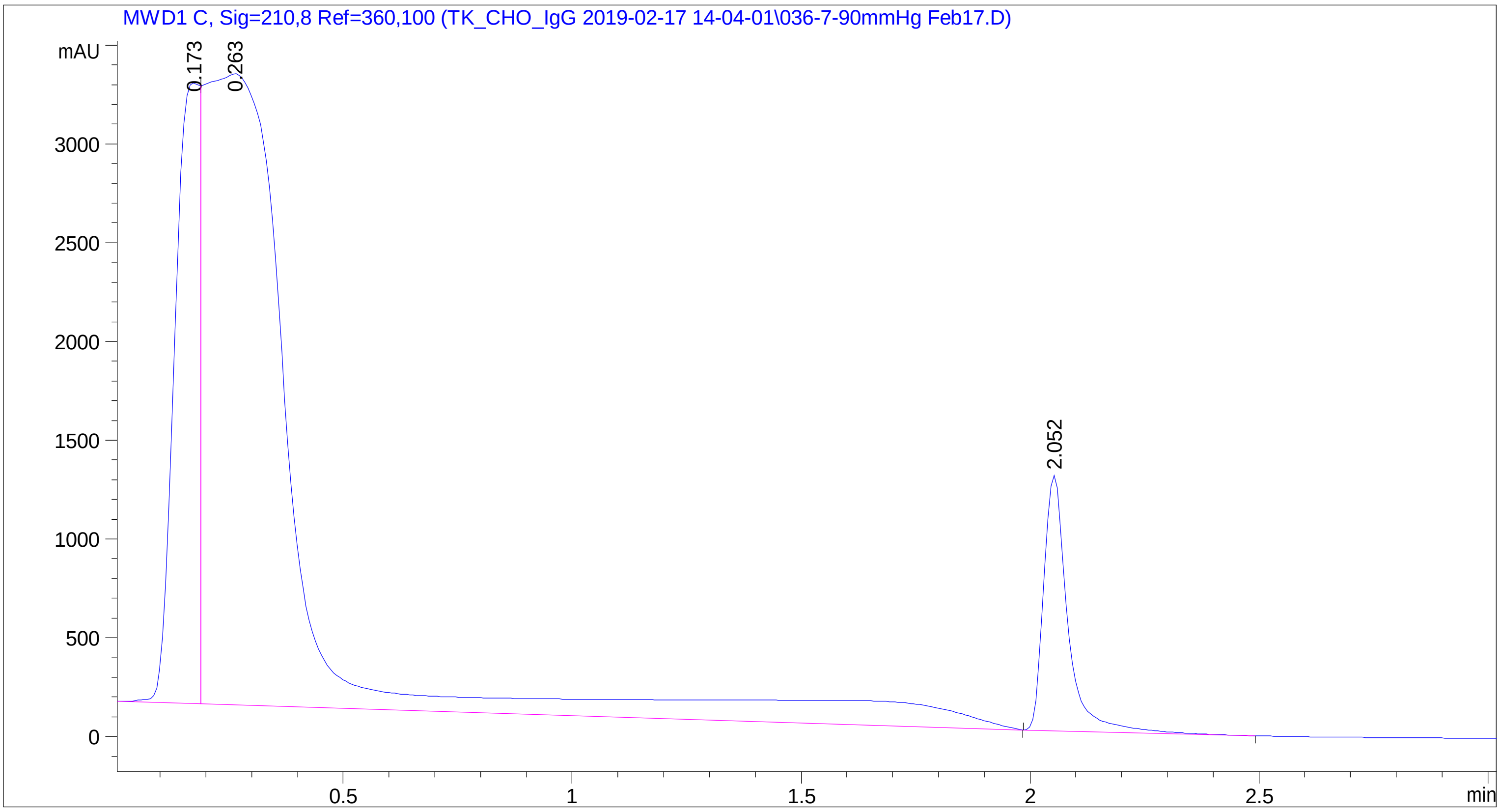

===
Area Percent Report
===

Sorted By : Signal
Multiplier : 1.0000
Dilution : 1.0000
Use Multiplier & Dilution Factor with ISTDs

Signal 1: MWD1 C, Sig=210,8 Ref=360,100

Peak #	RetTime [min]	Type	Width [min]	Area [mAU*s]	Height [mAU]	Area %
1	0.173	BV	0.0596	1.17557e4	3142.37964	19.4067
2	0.263	VB	0.1949	4.40620e4	3195.76245	72.7390
3	2.052	BBA	0.0549	4757.75684	1292.90369	7.8543

Totals : 6.05755e4 7631.04578

===
*** End of Report ***
